# Appearances on Dissection of Col. *******, M. P. Who Lately Died at Leghorn; with Observations

**Published:** 1816-09

**Authors:** 

**Affiliations:** an Italian Physician, who attended him in his last illness


*Appearances on Dissection of Col.* *******, *M. P. who lately died at Leghorn; with Observations.* By Dr. PALLONI, an Italian Physician, who attended him in his last illness.

THE Gentleman, who forms the subject of the following paper, was a public character of distinction, and a Member of the British Legislature. He was accompanied to the Continent by a medical gentleman of great talents, and both the physician and patient were well aware of the nature of the disease they had to combat. It was evidently aneurism, or some great organic derangement of the heart; but by the unwearied attention of his medical friend, and the most rigid regimen and caution on the part of the patient, the fatal catastrophe was warded off for a most unusual period of time, and until such a series of *effects* in other organs and parts of the body, was induced, as must excite the astonishment of every pathologist who reads this case.

Leghorn, April 5, 1816.

*Sectio cadaveris.*—"The external parts of the body presented nothing worthy of remark, excepting considerable emaciation and flaccidity.

"On inspection of the thoracic viscera, the following derangements were discovered, viz.

"*Primo.* An extravasation of about three pounds of pure blood in the two cavities of the thorax, proceeding from the rupture of some vessels on the surface of the lungs.

"*Secundo.* The pleura inflamed, thickened, and indurated; the pulmonic reflection adhering pretty generally to the costal, except where the extravasated blood was situated.

"*Tertio.* The lungs were of a livid colour from an extraordinary congestion of blood; but, in other respects, their texture appeared natural.

"*Quarto.* The pericardium was inflamed, thickened, indurated; and contained a small quantity of extravasated fluid in its cavity.

"*Quinto.* The heart was at least double its natural size, in consequence of a passive dilatation of its *right* auricle and ventricle, the parietes of which were thin and flaccid. The *left* auricle and ventricle were nearly in a natural state.

" *Sexto.* A large polypus was found in each ventricle of the heart, which, in other respects, was empty of blood.

" *Septimo.* The coats of the great arch of the aorta were thickened, and the calibre of the vessel contracted, particularly about the origins of the subclavian arteries, where the aorta was completely ossified.

" The diaphragm was natural. The stomach much distended with air; and the vessels on its left curvature were injected, and dilated to the size of a crow-quill. Its corresponding internal membrane was inflamed, and, in some places, gangrenous.

" The general cavity of the abdomen contained a small quantity of extravasated fluid, which probably was effused immediately before death. The liver was larger than natural, but no particular organic derangement could be discovered. Into the substance of the spleen a quantity of blood was poured, and its inferior edge was slightly inflamed.

" The kidnies were so disorganized, and their natural structure so entirely changed, that their ordinary figure, colour, and consistence were no longer cognizable. They were converted into a whitish pulpy mass, (en une blanchante Bouillié) in the middle of which, some small calculi were found. The emulgent vessels alone remained unaltered. The ureters and urinary bladder were natural, except the neck of the *latter*; and also the prostate gland, which were enlarged, thickened, and indurated.

" The omentum, mesentery, peritoneum, and all its continuations partook of the changes which we have stated to have taken place in the pleura:—that is, they were all injected, as it were, with blood; thickened, and in several places adherent to the contiguous parts, to one another, and to the neighbouring parietes.

OBSERVATIONS BY DR. PALLONI.

" From this inspection of the body, and from an analysis of the organic derangements which we have related, it may be allowable to infer,

" 1mo. That the most essential disease, and perhaps the primary cause of all the other phænomena, consisted in the morbid thickening of the coats of the great arch of the aorta, and in the diminution of its calibre at the ossified portion, where the subclavian arteries are given off.

" 2do. That from this aortic stricture, the column of blood propelled by each systole of the heart into the arch, would meet with resistance, and an impediment to its free course; and that, while retrograding, it would occasion an obstruction in the *pulmonary* circulation, and ultimately give rise to the dilatation of the *right* auricle and ventricle of the heart: hence the dreadful palpitations; the difficulty of respiration on taking exercise; the oppressed, irregular, and intermitting pulse, with which the patient laboured during so long a period; and hence, finally, the polypi in the ventricles of the heart itself.

"3tio. That this cause, continually increasing, *pari passu*, with the weakness of the powers of the heart, in consequence of its passive dilatation, occasioned such slowness and irregularity in the general circulation as ultimately gave rise to a chronic state of inflammation throughout all the membranes and viscera of the body, and particularly in the membranes of the heart and lungs.

"4to. That the immediate cause of death was the extravasation of blood into the cavities of the chest, from the rupture of some of the pulmonary vessels immoderately distended with the vital fluid.

"5to. That the very uncommon change of structure in the kidnies proceeded from the same cause, i. e. chronic inflammation, which they had suffered in common with the other viscera; and that, since the secretion of urine in this gentleman's case had never failed, nor even appeared at all affected, to the very period of dissolution, we may, with great probability of truth, conclude, that *this* secretion does not require that integrity of structure in the kidnies, which other secretions do in their respective glandular filtres, because the urine does not demand a proportionate degree of elaboration during secretion; but on the contrary, that, in the present and similar cases, urinary secretion was performed by the extremities or inorganic pores of the emulgent arteries.

"6to. If I were not fearful of being accused of presumption in endeavouring to trace the *first origin* of these organic diseases of so many important viscera, I would venture to say that it sprung from a hereditary gouty diathesis, which, instead of fixing itself in the upper or lower extremities, the usual seats of the disease, had been *translated* to the internal membranes, producing in them chronic inflammation, and that alteration and ossification of the aorta which have been already described.

"7mo. Finally, from an attentive reflection on these organic lesions, we cannot but allow that the valuable life of the ******** has been prolonged as much as possible by the continued care and judicious prescriptions of the learned and ingenious professor who accompanied him; and who, well aware of the aortic ossification, and organic disease of the heart, always endeavoured to proportion the mass of blood in circulation to the feeble contractions of the heart, and the obstructed channels through which it flowed; while, at the same time, he combated the chronic inflammation of the viscera and membranes, by spare diet, moderate exercise, the occasional exhibition of laxatives, and repeated bleedings. These means would probably have prolonged his existence still farther, had not the unfortunate rupture of some blood-vessels in the lungs, during his last illness, occasioned that fatal hæmorrhage which rendered every remedy unavailing."

(Signed) " DOCTOR PALLONI.

" *Leghorn, 10th April,* 1816."

---

### OBSERVATIONS BY THE EDITORS.

We conceive, that although the more prominent symptoms during life are only glanced at in the foregoing communication, yet that it possesses great interest, and is calculated to afford materials for useful reflection. We are not, at this day, to learn the symptoms which denote passive aneurism of the heart, or ossification of the aorta; but it is probable, that we did not before know how far these dreadful, and ultimately fatal lesions, are under the control of medicine and regimen; and how long life, and even some degree of enjoyment, may be lengthened out under accumulating evils, the progress of which we can retard, but not arrest. If it be said, that existence is scarcely worth prolongation, when accompanied by such miseries, and when under such constant dread and danger of termination; we answer, that we live not for ourselves alone. Our families, our friends, our fortunes, may be materially influenced or affected by a year's or even a month's procrastination of our fate. But, independently of these considerations, Nature herself so abhors the tomb, that there is hardly any species or degree of pain which she will not endure, rather than—

" —— leave the warm precincts of the cheerful day,"

and mingle with that mould from whence the Almighty *fiat* first caused her to start into existence!

The pathological views of our Italian contemporary on the above case, are by no means uninteresting: but although we cannot for a moment doubt, that the aortic stricture *indirectly* produced the dilatation of the *pulmonic* cavities of the heart, yet it is not easy to explain why the *left* side of the heart did not *first* suffer, as it must certainly have *first* felt the effects of the aortic obstruction. Was it because the left side of the heart is naturally stronger than the right, and consequently less disposed to passive dilatation, when obstruction is offered to the issue of blood from its cavities?

Dr. Palloni's idea of *translated gout* giving rise to the chronic visceral and membranous inflammation, may not accord with the views of some classes of the profession; but we are disposed to think, that the doctrines and opinions of a modern pathologist,* whose acuteness, discrimination, and unlimited experience, have raised him to the height of medical reputation, will have a powerful influence on the opinions and practice of the faculty at large; and the late work of that excellent physician certainly countenances the hypothesis of our Italian friend. We believe that both the *repellent* and *expellent* systems for the cure of gout are verging fast to a natural death; or, in other words, that the application of *cold water* to the extremities, and "*medicinal water*" to the stomach, will not have an *unprejudced* advocate in the course of five years.

This gentleman's life was preserved for *several years* after enlargement of the heart was quite manifest; and probably his span of existence might have been still farther protracted, had it not been for the effusion of blood in the lungs. The state of the different viscera, but particularly of the kidnies, offers an instructive lesson on the beneficial effects of *abstinence and evacuations*, in what might be termed desperate and fatal disorganizations of structure. There cannot be a doubt, but that, independently of the disease in the heart and aorta, the other visceral lesions would have destroyed life many years before, under the usual routine of treatment pursued in chronic complaints; and consequently that to the *cardiac* symptoms, the patient probably was indebted for the procrastination of his fate. EDIT.

---

* Dr. Parry, of Bath.

